# Spirit of the Foreign Periodicals, &c.

**Published:** 1836-07-01

**Authors:** 


### Spirit of the Foreign Periodicals, &c.

### Hints respecting the Changes which the Urine undergoes in Disease, and the Therapeutic Indications to be derived from these Changes.

When we consider that the chief, nay rather the sole and exclusive end, which the urinary secretion serves in the animal economy, is to carry off the effete and foreign matters of the system, and thus to purify the blood, (and if the blood, then every part, solid as well as fluid, of the living machine), we are prepared to expect that a right condition of this function must be necessary to the continuance of health, and that all deviations from health will probably be accompanied with some derangement of the qualities of the urine.

There was a time when the study of this fluid in diseases was carried to an extravagant extent; when doctors pretended to divine not only the type or general character, but even the exact seat of existing diseases, by merely inspecting the urine, and when they founded their treatment solely on the data furnished by such an examination. This, like every other exclusive and one-sided method of pathological inquiry, must of a necessity be erroneous, and has long been obsolete. We believe that there is only one lineal descendant of the "urine-doctors" now existing, in the person of a noted quack in some part of this huge metropolis, who has for many a year reaped a golden harvest from the well known credulity of our countrymen.

That however the study of the changes which the urinary secretion undergoes during diseases, deserves a more studious attention, than of late years has been bestowed upon it, we think will be admitted by all who bear in mind the physiological functions which it fulfils in the animal economy, and the important therapeutic aid, which an examination of its qualities has already afforded in the treatment of calculous disorders. We speak from multiplied experience, when we assure our readers, that in many other disorders, as well as in the calculous, the observation of the properties of the urine will frequently assist them much in determining the appropriate remedies and diet. In the treatment of all fevers, and more especially in the slow protracted or remittent fevers of children and young people, we have observed that the condition of the urine is one of the surest and most steady tests of the persistance, aggravation, or abatement of the masked febrile symptoms. The secretion is invariably scanty, deep coloured, and sedimentary, and of a strong smell during the early stages of the disease, while the tongue is furred, the alvine evacuations are unhealthy and offensive, the skin dry, and the pulse hurried and sharp. One of the earliest signs of an abatement of the fever, and one of the surest signs of the recovery of our patient, is the increased flow, and the improved appearance of the urinary discharge. The tongue will be found at the same time to be moist, and beginning to clean; the skin will no longer be harsh as hitherto, but soft and pliant; the pulse may still be rapid, but it will be found to be soft, and compressible; and there will probably be some indication of returning appetite. It may be said, that as the change in the urine is not a precursory, but merely a coincident phenomenon, it cannot afford much assistance in directing the treatment of such a case. This is quite true, and all that we are arguing for is, that the state of the urine deserves to be examined by the physician, as well as the state of other parts, and of other secretions. No sensible practitioner now-a-days omits to ascertain for himself the condition of the alvine evacuations, and he no longer trusts to the vague and incorrect reports of attendants. The benefits which have been derived from this practice are incalculable. Indeed it is downright empiricism to treat many diseases, especially fevers and that host of disorders which are associated together under the convenient appellation of liver complaints, stomach complaints, indigestion, &c. without a frequent examination of the stools; and a little experience will enable the attentive observer to distinguish the abnormal secretions, produced by disease, from those which are induced by the operation of purgative medicines.

Of almost equal importance is the examination of the urine in many of such cases, not only by affording direct indications of the state of the system at the time, but also by enabling us to correct some of the fallacies which exclusive attention to the alvine evacuations is occasionally apt to promote. Whenever the urine is scanty, deep coloured and sedimentary, and the stools are at the same time offensive and discoloured, it is a safe and valuable rule of practice to exhibit aperients conjoined with some of the saline diuretics,* to withhold all strong, greasy, stimulating food and drink, and to employ in their stead light animal broths, farinaceous compounds, and slightly alcaline beverages, by far the best of which is potash, or soda-water. The marked success of the use of these simple expedients in many protracted cases of dyspepsia and general derangement of the whole system, can only be duly appreciated by those who have witnessed or have experienced the speedy relief which they so often afford. In the treatment of such cases the physician will derive the most valuable assistance from a frequent appeal to the state of the urine.

Often indeed he is precluded by many circumstances from ascertaining the condition of the alvine motions, and he is obliged to trust to the misleading reports of his patient, who will assure him of their perfect healthiness, when they are most unnatural; the tongue too at the same time may appear clean and otherwise normal; the appetite may be moderately good or even unusually keen, and the physician may be thus misled by supposing that every thing is going on quite well, and that all the functions are recovering a healthy tone. Such an error may very generally be avoided by a reference to the state of the urinary secretion. Patients are more in the habit of observing the state of their urine, than of their stools; and the medical man may often ascertain the state of the one,

* As a general rule, none is better than a calomel pill taken at bed-time, and the infusum sennæ with tartrate of soda on the following morning. When this draught is not sufficiently active, a few grains of jalap powder added to it, will invariably answer the desired purpose. This combination is certainly one of the best hydragogue purgatives.

when he cannot do that of the other. If the urine be found to be deficient in quantity, of a deep colour, strong offensive smell, if it is cloudy and disposed to separate the uric acid sediment, he may be quite assured that the organs of digestion and chymification are still at fault, and that this fault consists, in nine cases out of ten, in a diseased condition of the intestinal secretions, and probably in an accumulation of unhealthy fæces in some part of the bowels. We admit indeed that this rule, like every other rule of practice, is to be received "cum grano salis." At certain, especially the advanced, periods of life, there is sometimes a very considerable excess of uric acid in the urine, and a strong tendency to its sedimentary deposit; and along with this state of the secretion, the quantity of it may be greatly diminished, and yet no decided derangement of the general health may be the consequence; but such an occurrence is only occasional even in old age, and certainly it is of extreme rarity in youth and middle age.

The diseases, in which the changes alluded to of the urinary secretion are most conspicuous, and in the treatment of which we have derived the most obvious advantages from attending to these changes, are the various forms of indigestion, in all obstinate non-inflammatory intestinal disorders, in gout, and some of the varieties of chronic rheumatism, in asthma and in cutaneous affections.

In cases of obstinate dyspepsia, the urine is never quite healthy, at least for any considerable length of time. When it is deranged, the alteration very generally consists in an excess of the uric or lithic acid.

We have, times out of number, remarked that after every aggravation of the stomach ailments, however slight and neglected, the state of the urine was almost invariably more disturbed than it had been immediately before the aggravation; and thus from merely examining the urine, we have often been able to predicate our patient's condition, before making a single inquiry. In this way too we are often enabled to judge of the beneficial effects of the medicines and the diet which we may have prescribed; and we are thus furnished with at least one method of shrewdly suspecting whether our prescriptions have been faithfully obeyed or not. It is to be kept in mind that in the cases, to which our present remarks refer, we are supposing that the state of the urine has been deranged for a considerable length of time.

The merely occasional alterations of this excretion, although deserving of attention as indicative of some stomach or intestinal annoyance, seldom demand professional relief; and when they do, a few doses of an active purgative, with restriction of the diet for a day or two, generally suffice to remove them. The more protracted cases require a nice and delicate management. Practical experience has shewn us that much valuable assistance in the treatment of dyspeptic disorders may be derived from observing the state of the urine. Hitherto there has been far too much of mere empiricism in this department of therapeutics. Medical men have followed no steady nor rational principles in guiding their selection of the appropriate remedies. For example we find among the numerous list of stomachic and antidyspeptic remedies, both acids and alcalis enumerated; and to the best of our recollection, there have never been any directions proposed to assist the medical man, in determining which of these very opposite medicines he should employ in any particular case. One physician may recommend the sulphuric or the muriatic acid, either alone, or in combination with a bitter infusion, while another, under the very same circumstances, will prescribe the carbonate of potash, or of soda, magnesia, or some other alcaline preparation. Now although we are by no means addicted to the chemical reasonings of the older physicians with respect to the operation of all remedies, we really cannot enlarge our credulity so far, as to believe, like the well-meaning Vicar, "that much may be said on both sides of the question" in the present instance of medical incongruity. That acids are excellent remedies in many cases of dyspepsia, we are quite ready to admit; for no one who has employed them with discrimination can possibly be of a different opinion; and the same qualified praise is due to alcaline preparations also; but we cannot for a moment allow that acids and alcalis can be equally useful in the same case, or set of cases.

In the dyspepsia of old, infirm and worn-out constitutions, when there is no gastritic or enteritic affection present, and when the alvine discharges have been brought into as healthy a condition as possible, it will generally be found that the urine is either pale and clear, or that it is ropy and has a tendency to a white chalk-like deposit.

It is in these cases that the use of the mineral acids is so strikingly beneficial: the appetite is improved by their use, the feelings of general comfort are agreeably changed from languor and despondency to comparative strength and cheerfulness, and with these changes the urinary secretion is brought back to a healthy state. On the other hand, whenever the urine is scanty, deep coloured, and deposits the lateritious sediment, as is very generally the case in the dyspepsia of younger and more robust persons, the use of acids is improper, and advantage will almost certainly be derived from the judicious use of alcaline remedies.

In order that the diagnosis of the chemical changes of the urine may be more correct, the employment of litmus and turmeric paper, as a means of testing its acidity or alcalinity, ought never to be neglected.*

The increased acidity is, it is well known, coincident with the deposition of the red lateritious sediment; and the diminished acidity, with the deposition of the white sediment. These few hints will be sufficient to explain our views of the importance of attending to the state of the urine in dyspeptic complaints: they may possibly excite a train of enquiry on the part of the scientific reader. Hitherto we have spoken only of dyspepsia; but the remarks which have been made in reference to this disease, are strictly applicable to gouty and rheumatic complaints, and to many cases of asthma, and also of cutaneous disease. In truth dyspepsia is almost always an adjunct, nay sometimes it is the actual cause of these very disorders; and hence on the successful treatment of the stomach and bowel affections, will the cure of the more prominent illness be often found to depend.

When the symptoms of indigestion are well marked, as is usually the case in gout and often in asthma, the attention of the physician is necessarily attracted to the state of the stomach, and he is thus fortunately led to employ the appropriate remedies.

It may be worth while to notice here a fact, which in a certain degree seems to indicate some connexion, or at least co-existence of gouty indisposition with irregularities of the urinary secretion, that the very remedy which has so deservedly acquired great repute for the cure of the gout has a marked influence on the state of the urine, by increasing the quantity of its uric acid. How it effects this, we do not presume to say; but we believe that it is very generally admitted by writers on the materia medica that, while the system is under the influence of colchicum, there is an unusual quantity of the uric acid contained in the urine.

In cases of asthma, the paroxysm is often preceded and accompanied by a marked diminution in the quantity of the urine; as the paroxysm abates, the quantity of the urine gradually increases, and it is at the same time usually high-coloured and deposits a large quantity of red sediment. How often it is that a fit of asthma appears to terminate in a copious diuresis!

The other forms of subacute disease in which we have observed that the urine is very generally deranged, are chronic rheumatism and many skin

* Litmus paper is reddened by acid, and is not affected by alcaline urine; turmeric paper is stained brown by alcaline, and is not affected by acid urine.

complaints. We are the more anxious to direct the reader's attention to these, because hitherto the state of the urinary secretion has been so much neglected in the descriptions of modern writers.

That there are many cases of chronic rheumatism and of skin disease in which the urine is never, or is only casually abnormal, is quite true; but as the reverse of this position is as often so, it is the duty of the wise physician to observe the state of this important secretion in all cases without exception. In chronic rheumatism, especially when the pains are erratic, and when the constitution of the patient is what is usually termed "bilious," it will be found that the urine seldom remains for any length of time healthy in quantity and quality, and that almost invariably whenever the pains are most severe, the changes in the urine are most conspicuous.

The intestinal secretions may be disturbed at the same time; and even when they are not very sensibly so, we may be still assured that the stomach and bowels are at least prone to occasional derangements. There is an error which those medical men, who regard the vigour of the appetite as not an unfair criterion of the energies of the stomach, are apt to fall into; it is that of giving their patients a carte-blanche to eat of whatever food, they believe, does not disagree with them. Now in not a few instances of indigestion, such an agreeable permission is decidedly injudicious. Often, very often, the appetite is as strong, or even stronger than in ordinary health, and yet the digestive and assimilating powers of the stomach and bowels are unquestionably weak and depraved: otherwise how should the body become emaciated and enfeebled, while abundance of nutriment is taken, and the digestion is *apparently* healthy. At the present moment we have two cases under treatment, which illustrate these positions admirably well. A military officer had, at the period of our first visit, been laid up for four months with a most troublesome rheumatic affection of the hip-joint. The affection was deemed rheumatic in consequence of his having repeatedly suffered from similar attacks in other joints, and of the pain and local distress being greatly influenced by atmospheric changes. He had been under the treatment of an hospital surgeon of this metropolis from the commencement of his illness. Caustic issues had been made over different parts of the joint, and an occasional purgative with colchicum administered; but as the general health of the patient seemed to be not all affected, his appetite being good, and the bowels acting regularly, little regard had been paid to constitutional remedies.

As the local malady however seemed to be as obstinate as ever, he requested me to give him my opinion of his case. Upon enquiring of him what was the state of the urine, he told me that he had never noticed it. I found that it was scanty, deep coloured, and that on resting it deposited a quantity of lateritious sediment. The alvine evacuations also were darker, and decidedly more offensive than in health. By the use of purgatives first, and then of alcalis combined with colchicum, and by the appropriate regulation of his diet, this gentleman's health was speedily improved; and afterwards by the exhibition of small doses of the corrosive sublimate, so as to affect the system with the mercurial action gently, the cure was made complete.

The other case is still more apposite. A middle-aged gentleman, of a phlegmatic bilious constitution, and rather addicted to the pleasures of the table, had for four years suffered from general derangement of his health. He was subject to violent headaches, to severe wandering pains affecting chiefly the thighs, legs and feet, and lasting usually from six to twelve hours, to occasional attacks of syncope, and to universal languor and depression. His complexion was muddy and every now and then he looked half-jaundiced.—His appetite however was always vigorous, and often quite craving. His bowels acted, he said, regularly, and as to his urine he had paid no attention to it. He had been treated by at least a dozen doctors in London, he had visited also some of the fashionable watering places, and we may therefore suppose, a multitude of remedies had been tried.

I was surprised to hear that not one of his medical attendants had ever taken the trouble to ascertain for themselves the state of the alvine and urinary secretions.

On examination the stools, after the operation of an active purgative, were found to be of a dark green colour, slimy, very fetid; and the urine was small in quantity and very sedimentary. The use of purgatives was persevered in steadily for several days, until the motions became of a healthier aspect, and had lost their extreme offensiveness. He then took alcalis combined with colchicum for two or three weeks, and all malt liquors, acid wines, greasy, and oppressive food were interdicted. The quantity of the urine was thereby greatly increased and it became gradually quite clear.

By a few simple instructions which I had given him, he was readily able to judge for himself of the healthiness or unhealthiness of the stools and urine; and he soon discovered that invariably after each attack of pain the urine was thick and deposited a lateritious sediment, and that whenever the appearance of the stools deviated from health, his feelings of general malaise were always more decided. The treatment throughout was simple and ultimately efficacious. Pills consisting of equal parts of extract. colocynth. c., the extract. colchici, and of pil. hydrargyri, were taken every, or every second, night; and minute doses of the oxymuriate of mercury dissolved in the compound tincture of cinnamon were taken twice daily for three or four weeks at a time; then omitted for some days, and again resumed.

This treatment will be found admirably suited to such cases, provided the bowels and kidneys have been previously brought into a healthy condition, and all occasional irregularities are immediately corrected. As to diet, the lighter foods must be preferred; and as to drinks, none assuredly is so good as soda or potash water, either alone or with small quantities of old sherry wine, or of brandy, or hollands.

Our limits prevent us from discussing at present the connexion between cutaneous diseases and irregularities of the urine. Our readers will find a few remarks on this highly important and much neglected subject, in our review of Rayer's great work in the number for last October.

In all cases of skin disease, and especially in those of a chronic nature, the urine ought to be examined and tested. We may be quite assured that the functions of the skin are never performed quite healthily, when the urinary secretion is depraved.

We are not provided with sufficient data to authorize us in laying down any very definite instructions, derivable from the condition of the urine, to guide the physician in the treatment of cutaneous diseases. The subject is one of great therapeutic interest, and we therefore earnestly invite all dermatologists to investigate it with attention. Perhaps there is not a class of diseases, in the cure of which our remedies are more frequently nugatory, than in those which appertain to the skin. Many improvements have been of late years introduced, and the medical man is on the whole not so much embarrassed and so often baffled as he was wont to be; but still cases, apparently the most simple, and involving no structural changes of the skin, as lichen, prurigo, eczema, &c., are occurring every day, where the ordinarily-recommended remedies are utterly impotent. It is quite probable that a more accurate knowledge of the chemical state of some of the excretions might lead to a more uniformly successful practice.

Before quitting the subject which has engaged us in the preceding pages, it may be useful briefly to allude to some of the more obvious and remarkable changes in the urine, which are indicative of, or at least attend certain other morbid states of the body. In hysteria and in all diseases dependent upon undue excitability of the nervous system, the urine is very usually much increased in quantity, and it is at the same time exceedingly pale and limpid. This condition of the urine is common also in the climacteric decay of the body, as old age comes on. The kidney is in many such cases the organ which is soonest and most distinctly affected; and hence whenever we find that the vital and animal energies are becoming gradually more and more enfeebled, without any apparent cause or local suffering, the state of the urinary secretion should be assiduously watched. Diabetes insipidus is very frequently the forerunner of a general "breaking up" of the system. Even at the earlier periods of life, especially when the constitution has been enfeebled by irregularities in living, by debauchery, excess of venery, over-violent exercise, &c. an increased flow of the urine (in such cases it very frequently contains a larger quantity of solid ingredients, than in health) precedes and indeed causes an irremediable exhaustion of the powers of life. As a general remark, we should advise the medical man to have his attention directed to the urinary secretion, whenever there is a gradual and increasing emaciation of the body without any prominent existing malady.

That state of the urine which has been denominated the phosphatic state, in which there is a greater or less tendency to the deposition of the phosphates of lime and of magnesia, and in which there is a corresponding deficiency of the uric acid, is of frequent occurrence in scrofulous unhealthy children, especially whenever any mesenteric disease is present; also in advanced age, if the powers of digestion and of assimilation are enfeebled or depraved; in hemiplegia and after all severe accidents to the spine; and lastly, in all chronic diseases of the kidneys and bladder.

Albuminous urine, if it has been of long duration, is usually symptomatic of some visceral disease, more especially of disease of the kidney (dropsy being frequently the consequence), and also, although less frequently, of the liver. Occasionally it is found to be coexistent with merely temporary derangements of the digestive and respiratory functions, when no organic mischief is present in any part. In the dropsy which succeeds to scarlatina, and in all dropsies when the effusion has been preceded by inflammation of the respiratory organs, or when there is co-existent disease of the heart, the urine is often albuminous.

Although this state of the urine, therefore, may unquestionably exist without organic disease of the kidneys being present, yet when it has continued for a considerable length of time, and the system is gradually decaying, and yet does not manifest symptoms of inflammatory distress, there are strong grounds to excite the suspicions of the physician as to the state of the kidneys, whether dropsical symptoms be present or not. The late Dr. Darwall seems to have been of opinion that the coagulability of the urine is not often dependent upon that form of organic disease of the kidneys (which, it has been proved by the researches of Drs. Bright, Christison, Gregory and others, is so frequently accompanied by dropsical effusions), but rather upon some previous or coexistent disturbance of the respiratory functions; and he suggests that the changes in its chemical properties is probably owing to the blood which is conveyed to the renal organs, having been imperfectly changed and purified during its passage through the lungs, in consequence of the partial obstruction or impediment to their free play from disease, either of their own texture, or of the heart. The opinion of Dr. Blackall, that a coagulable state of the urine is indicative of inflammatory action, and therefore demands the use of antiphlogistic treatment, is only partially and occasionally true. Dr. Bright and other practical physicians have recorded several cases of dropsy in which the urine was coagulable, and in which nevertheless steel and other tonics proved decidedly useful. The urine has been often observed to become coagulable, when the system has been affected with mercury. From all these considerations we are led to agree with the opinion of that excellent chemist Dr. Prout, who, in speaking of the value of the coagulability of the urine as a means of diagnosis, remarks, that we ought always to be aware of its presence, as taken along with other symptoms it may be occasionally useful in directing our judgment of the nature of the disease, but that, in the present state of our knowledge, it does not indicate any particular remedy, or mode of treatment.

To detect the presence of albumen in urine, we have recourse either to boiling, to nitric acid, to the ferrocyanate of potash (adding previously a few drops of acitic acid) or to corrosive sublimate.

As we are at present on the subject of the analysis of the fluids in disease, we are tempted to introduce a few cursory remarks from a short work recently published by Mr. Rees. He mentions that Dr. Marcet found in the blood of a diabetic patient so much fat, that the serum was like an emulsion in appearance and deposited a substance like cream : it putrefied very quickly.

The foreign principles occasionally detected in the blood are urea (in cholera and in some sorts of dropsy attended with a coagulable state of the urine); cholesterine and the colouring matter of the bile (in jaundice and some other diseases) ; perhaps also sugar and free carbon : but the presence of these two last are of more doubtful authenticity. M. Lecanu has shewn that the serum of the blood in cases of jaundice contains occasionally the following abnormal ingredients : a combination of albumen and soda, scarcely at all soluble in water ; an orange yellow colouring principle ; and a blue colouring principle. These colouring principles exist normally in the bile.

The yellow serum of the blood in cases of jaundice may be tested for bile by adding dilute sulphuric acid to it :—the colour is changed after a few minutes to a delicate green colour.

In reference to the changes of the urine, it may be worth while adding to what has already been stated, that any vegetable colouring matter, which may happen to exist in this fluid, is readily detected by adding caustic potash :—the urine immediately assumes a green colour. Mr. Rees mentions a case of suspected hæmaturia : the dark red colour was owing however to the urine being tinged with beet-root, which the patient had eaten in a salad.

Mr. Rees gives the following instructions to discover the pressure of iodine in urine. Evaporate the urine to dryness ; dissolve the residue in distilled water and filter the solution ; then add one eighth part of strong sulphuric acid. By adding now a solution of starch, the rich blue colour is immediately manifested. The presence of iodine may frequently be detected by simply mixing the urine to be tested with one eighth part of the strong sulphuric acid, and then suspending over the fluid a piece of blotting paper which has had a solution of starch dried upon it.

Dr. A. T. Thomson has recently suggested the following improvement of the process to detect iodine or any of its preparations in urine or in any other fluid. To the fluid to be examined add a small quantity of the solution of starch, and then a few drops of nitro-muriatic, or of nitrous acid. The blue colour is immediately manifested. We must be careful not to add too much of the acid, as an excess will at once dissolve the blue precipitate. Dr. T. mentions, that by this simple process he has detected the presence of iodine in the urine, the saliva, the matter of perspiration, as well as in tea, malt-liquors, &c.

---

### Detection of Sulphate of Quinine in Urine.

M. Piorry, one of the Clinical physicians of the Hôtel Dieu, has published the following novel observations on this subject. " Finding that the urine of patients who were taking quinine was sensibly bitter, I requested M. M. Vallée and Fermond to analyse it. The subjoined is their report. The urine of several patients, who had taken about half an hour previously a large dose of quinine, we acidulated with diluted sulphuric acid, and then evaporated the fluid to three-fourths of its original quantity. When cool, it was filtered, and then treated with quick-lime in powder, in order to decompose the sulphate of quinine and reduce it to the state of the simple alcaloid, which was precipitated along with the insoluble sulphate of lime. The precipitate collected on a filter was well washed, then dried and reduced to a fine powder. This powder was treated with alcohol, and allowed to digest for twenty-four hours: it was then filtered and evaporated, and the residue was treated with water accidulated with sulphuric acid: the solution gradually deposited a crystallised sulphate of quinine, which was readily recognisable by its well-known characters."—*Bulletin Clinique.*

---

### OBSERVATIONS ON SOME OF THE FORMS OF MONOMANIA.

To M. M. Pinel, Esquirol and Georget is due the great merit of having first clearly illustrated that very melancholy form of insanity, in which the mind is the victim of one particular delusion, or of one inordinate and extravagant passion or propensity, retaining its other intellectual capacities and moral emotions intact or nearly so. Of this partial insanity, to which M. Esquirol gave the name of monomania, there are two sorts or varieties,—according as the disorder is, or is not, attended with a disturbance of the judgment or reasoning principle. These two varieties have been distinguished by the appellations, "monomanie raisonnante," and "monomanie instinctive." The propriety of this distinction has been recognized by the greater number of recent medical jurists of Germany. M. Henke, who has hitherto denied the existence of an instinctive monomania in its full and absolute sense, assents however to all the facts which have been reported by authors as indicative of a special disease or insanity of the *will;* he admits that the patients in certain cases have not retained their freedom of agency, and could not therefore be regarded as responsible for their actions; "but," says he, "we recognize in this abnormal or morbid state of the affective faculties (the moral or active powers of English metaphysicians—*Rev.*) and of the will, only the antecedent condition (point de depart) of that derangement of the intellect or reason which occurs at the moment of perpetrating the deed,—a derangement which alone can excuse the violences to which the unhappy patients are sometimes driven. What distinguishes man, as a moral being, from the brute animals is, that he possesses a reasoning principle capable of regulating the instinctive impulses and the depraved desires which may spring up in his bosom: as long as this reasoning principle is not perverted or abolished, man must be considered as a responsible agent, whatever be the violence or intensity of the emotion which leads him to commit a criminal action.

If we observe men of a mild humane disposition and virtuous principles induced to commit the most extraordinary violences, such as attempting to murder their dearest friends or relatives, it is impossible not to believe that, in the moment of the act at least, there was some disturbance or derangement of the reasoning powers, although the patients immediately before and after the deed appeared to retain them in their full integrity."

M. Henke appeals to the doctrines of M. Esquirol in support of these views; but it appears to us that he has not quite correctly understood M. Esquirol's sentiments on this subject. M. E. does not restrict, as M. Henke supposes, the appellation "monomania" to a partial hallucination (delire) produced by a dominant and exclusive idea; but comprehends under this term also that form of insanity, in which no mental hallucination at all exists, but where the will alone is enthralled and subverted by an excessive and unconquerable feeling or emotion. In treating of the homicidal monomania, he says that there are two forms of it. In the one, the propensity to murder is provoked by a delusion of the reason arising, it may be, from a supposed insult or grievance, from a dread of personal injury and therefore in self-defence, or from misguided patriotic or religious convictions; whereas in the other we observe no appreciable disturbance of the intellect; the miserable person being led captive by a mere blind instinct, a *something* indefinable, which goads him on to murder and destroy. The former is the "monomanie raisonnante" and the latter is the "monomanie instinctive" of the French authors.

M. Amelung seems to have adopted the views of M. Henke in reference to monomania, and he is therefore unwilling to recognise the propriety of admitting an instinctive form of this insanity. He thinks that, in all such cases, the reason or judgment is positively and essentially deranged at the moment of committing any crime.

M. Hartmann entertains rather different sentiments. He says: "In the mania without delirium or mental hallucination, it is not from any illusory dreams of the imagination that the person is goaded on to the commission of insensate acts; but rather from certain diseased sensations, which have seized upon the mind, absorbing all its attention and preventing it from reflection upon any of its other relations. In this manner the reason, without being deranged or perverted, is, as it were, overpowered for a certain period."

Whichever view we take of this question, whether we suppose that there is necessarily an actual and positive derangement of the intellect or not, all medical jurists in Germany assent to the truth of the position, that "the will and the affective faculties may be so disturbed, as to induce, either directly, or indirectly by a temporary derangement of the reason, an entire abolition of the moral liberty, or free agency." It is upon this fundamental position that rests the modern doctrine of medical jurisprudence, relative to that form of monomania which has been designated the "instinctive." The essential point of this doctrine is the admission, that the moral liberty may be destroyed, not only by primary lesion of the intellectual or reasoning powers, but also by a special lesion of the mere affective or moral powers, while the reason *appears* to remain unaffected. We need scarcely say that this doctrine may be carried to a most unjustifiable extent by cunning and unprincipled men, and may be abused to the vile purpose of apologising for crime, and of exculpating the most profligate villains. It is of the highest consequence therefore to study this subject with the most conscientious attention, and endeavour to discover if there are any marks or tests which may enable us to distinguish the monomaniac from the responsible criminal. We propose, at the present time, to notice briefly the phenomena, physical as well as moral, which the German authors have observed in monomaniac patients, and afterwards to point out the relative value and importance which ought to be attached to these phenomena, as signs of the mental disease.

The persons in whom instinctive monomania has been most frequently observed are nervous, hysterical women, or those who have been recently delivered; hypochondriac, epileptic men; and lastly youths of either sex about the period of puberty. The patients exhibit almost invariably a more or less obvious degree of intestinal disorder, characterised chiefly by a constipated state of the bowels, and connected no doubt with an inactivity and tendency to congestion of the abdominal circulation. Very frequently there is a hæmorrhoidal disposition; the patient is subject to head-aches, and to other cerebral annoyances, as noises in the ears, dizziness, inaptitude for prolonged mental exercise, &c. and often he suffers much from a sense of uneasiness in the epigastric region. All these distressing feelings are aggravated by the sight, or even by the mere idea of the object of the monomaniac delusion. The sight of a knife, or stick, or firebrand is sometimes sufficient to bring on an ungovernable propensity to some act of mischief. The disorders observed in the psychological or mental functions are often quite as obvious as those in the body. The person is more or less gloomy and melancholy; he is quite conscious of his criminal propensity to stealing for example, or to murder, or to incendiarism, and yet he feels impelled on to their commission. At times he is so sensible of his unhappy condition, as to implore his attendants to withdraw every weapon of offence from within his reach. The gloom often finds relief for a time in bitter lamentations and weeping; and this frequently excites surprise in his friends, as they are perhaps not aware of the dreadful struggles of mind which the poor sufferer experiences.

Hitherto the reason has perhaps maintained the ascendancy: the vicious propensity is rejected with horror, and the patient, aware of his lamentable position, strives to extricate himself from it.

For example, a mother intrusts to the care of a stranger the child whom she tenderly loves. But, strange to say, the very dread which she entertains of her own possible actions, instead of weakening, often seems rather to exasperate her murderous propensity. She tries to reason with herself on the awful crime she meditates; perhaps she rushes to the altar of her God, and there implores divine grace to deliver her from the slavery of her passion, or she takes a distant voyage, to remove her from every chance of submitting to its power. Sometimes, however, in spite of all these efforts, the mischievous feeling becomes stronger and stronger; the idea of murder returns every moment with fresh energy, leaving her not a moment of repose, and the struggle between virtuous horror and the overpowering inclination becomes more and more painful.

If, at this unhappy moment, the object of her monomania is within her reach, the ferocious instinct, which neither reason, nor love, nor religion can control, may burst out with violence, and lead her on to the perpetration of the most dreadful enormity—the murder of her own offspring. The following case is reported by Wildberg, one of the most distinguished medical jurists of Germany. The patient was a gentleman, 51 years of age, of the most estimable character, and of high mental endowments. He was of a bilious nervous temperament, and had for a great number of years been subject to abdominal complaints, and to frequent and protracted headaches, owing, it would seem, to general venous plethora. His mind and temper were naturally lively and active, but he had become of late more and more hypochondriac, and the tendency to gloom had been aggravated by several domestic calamities. His literary occupations were now a source of annoyance and pain to him, and his restless anxiety was sometimes quite insupportable to his feelings. His eldest daughter, a girl 17 years of age, had been hitherto his greatest comfort and delight, and often would he request her to accompany him in his walks, that her cheerful conversation might dissipate his gloomy thoughts. One day, when this beloved child visited him in his study, the horrible thought of murdering her came into his mind. He was so dreadfully alarmed himself that he instantly told her to leave the room. When left alone, he wept bitterly, and it was long before his mind became tranquil. He resolved to take a short trip into the country, to compose his feelings, and to withdraw himself from the society of his daughter. Gloomy thoughts were in a great measure dissipated by this recreation, and he returned home more quiet; but no sooner did he see his child again, than the savage desire began to haunt him, and, in spite of the most painful struggles to resist its impulses, it increased in violence every day. M. Wildberg being consulted, strongly advised a separation of the daughter from her father's house, and recommended him a course of deobstruent purgative and antispasmodic medicines. In the course of a fortnight, this gentleman's health was very decidedly improved. His appetite returned, his sleep became quiet and refreshing, he felt altogether lighter and more comfortable, and his "moral" was greatly tranquillized. Occasionally he still felt a tendency to gloom and despondency, which, however, passed away in the course of a few minutes, and he would then join as freely as ever in the conversation of his friends. The same regimen was persevered in for another fortnight, and some tonic medicines were afterwards administered. The patient recovered a state of perfect health, and when his daughter was introduced to his presence, his joy, although chequered with the remembrance of his former feelings, exhibited all the ecstasies of a parent's fondness. Fortunately there was no subsequent relapse of the moral disease.

We now proceed to consider another feature of monomania; viz. the state of mind of the person immediately after the commission of any heinous act. The solution of this question is often of great importance in legal medicine. We may premise that the instinctive form of homicidal monomania, although its existence is now almost universally recognized, has very rarely led to the perpetration of murder. In the greater number of cases where murder has been committed during insanity, the mind of the person has been under the unhappy influence of a partial delirium, or specific hallucination, arising from some false or absurd idea which occupied the mind. These, therefore, were cases of "monomanie raisonnante," and not of "m. instinctive." Not so, however, with the incendiary monomania, which, 'per se,' has very frequently led to the commission of arson; the motive or impulse being merely a strong ungovernable propensity or instinct, and quite unconnected with any delusion of the intellect. In treating, therefore, of the state of mind in instinctive monomania, immediately after the perpetration of an offence, we shall have reference chiefly to cases of incendiary madness.

No sooner is the deed accomplished, than a calm succeeds to the state of violent agitation, such as we have described above. The unhappy person feels himself relieved, as it were, of a burden, and experiences a degree of comfort (bien-être) utterly unknown to those who have acted with criminal motives. The young incendiary, whose case is recorded by Klein, in the Annales Judiciaires, and by M. Marc, in the Annal. d'Hygiene for 1833, was in a perfect ecstasy of joy when she saw the flames burst forth. Very generally, however, these joyful feelings are only temporary—reflection is awakened, and gloom and remorse then occupy the mind. A young girl of a quiet inoffensive disposition, and whose character had been hitherto exemplary, made seven different attempts at incendiarism, in a village near Cologne. When interrogated as to the motives which had prompted her to act so wickedly, she burst into tears, confessing that, at certain periods, she felt her reason forsake her, and that then she was irresistibly impelled to the commission of a deed, of which, when done, she bitterly repented. She was acquitted by a jury of all criminal intentions.

From what has been now stated, it is easy to draw a very striking difference between such monomaniacs as we have been alluding to, and those who are labouring under a partial delirium or hallucination of the reason: the former very generally repent of the deed to which they have been driven—whereas the latter not only make no attempt to conceal it, but often glory in having committed it.

A much greater difficulty is experienced in attempting to point out any safe principles to guide the medical jurist, in distinguishing instinctive monomania from that state of mind in which a person, being goaded on by merely inordinate vicious propensities, yields to their impulses, and who must, therefore, be considered as responsible for the consequences of his actions. The question is, indeed, a very difficult one, and will often occasion a painful struggle between the compassionate dictates of humanity, and the rigorous demands of offended justice. Perhaps the following remarks may, in some degree at least, assist us in a right discrimination.

The circumstances of age and sex should be diligently noted. Very many incendiaries have been young persons, about the period of puberty, and perhaps, on the whole, girls have been more frequently implicated than boys. If the person has been subject to any nervous disorder, such as hypochondriasis, epilepsy, or hysteria, the medical jurist will bear in mind, that these morbid conditions favour the development of instinctive monomania. The puerperal state appears to have occasionally a similar influence in disturbing the mental functions. If, combined with any of the states now mentioned, there is a disposition to abdominal or cerebral irregularities, such as chronic affections of the intestinal canal, painful or deficient menstruation, headaches, &c. the German writers very properly attach considerable importance to these phenomena in their judicial reports. So much for certain physical or corporeal indispositions, the right appreciation of which should never be neglected in a doubtful case. But the scientific physician will not confine his attention to the consideration of these signs alone; he will also inquire into the morals of the accused; he will endeavour to find out his previous state of mind, his leading propensities and affections, the motives and temptations which may have prompted him to commit the offence, whether it has sprung from revenge, or lust of wealth, or any other sinful passion, and, lastly, the feelings which he displayed immediately and shortly after it was accomplished. In short, he must study "l'homme tout entier," in order to arrive at accurate and satisfactory results. We have not yet alluded to another feature in the case of the monomaniac who may have committed an offence—we mean, the utter want of precaution, either as to time or circumstances, in the execution of it. For example, no rational person would attempt to murder another in the presence of a number of people, nor to set fire to a house in open day, and when there were inmates in it. A girl, 17 years of age, became servant to a M. Becker on the 7th day of February. Strange to say, her master's house was discovered to be on fire several times in the course of a few days, after she began to reside there. The girl was dismissed, in consequence of her master supposing that she was bewitched. Soon afterwards she got a place in another family, and it was not long before she again resorted to her incendiary practices. When charged with the offence, she at once confessed it, and bitterly grieved at the damage and distress she had caused. The judge before whom she was tried very properly decided, that she was the victim of an instinctive monomania.

We propose now to consider briefly the other form of monomania—that in which the mind in occupied with a delusion or hallucination on one particular subject, or set of subjects, while on all other subjects it is perfectly sane. This is the "monomanie raisonnante," or "folie partielle" of the French medical jurists. Of late years, much attention has been paid to this very curious and puzzling theme, and many extraordinary cases of partial insanity have been recorded. We shall select a few from the writings of some of the German authors.

*Case* 1. A woman, who had for a long time suffered severely from domestic vexations, resolved to put an end to her existence. She had not, however, the fearlessness to commit suicide in a direct manner; but she set fire to a neighbour's house, and immediately confessed the act, in order that she might be apprehended, and condemned to execution. The question arose, whether this woman was to be considered insane or not. Several authors, in similar cases, have thought to determine the existence of monomania, by simply appealing to the very act, which they regard as the most complete proof of positive insanity. But this opinion is objected to, on the ground that the act may be, as in the present case, by no means absurd, either in point of design or of execution, and therefore that the derangement, if such does really exist, belongs rather to the antecedent state of mind which impels the person to self-destruction, than to the period at which the offence is committed.

This was the view of the woman's case taken by the physician who was charged with her examination. From minute inquiries he came to the conclusion, that she had been subject, not indeed to a confirmed monomania, but to gloomy melancholy, which must have interfered with the free exercise of her intellectual functions. He gave it therefore as his opinion, that the extreme rigour of the law should not be enforced against this woman; but that nevertheless she should be punished severely, as it could not be alleged in her favour that she had acted irrationally, or without premeditation.

*Case* 2. Platner relates the case of a young man who shot his companion, because he supposed that he was plotting against his life by witchcraft. He was quite well aware that he would be condemned to death; but, said he, "it is a thousand times better to die on the scaffold, than to perish miserably by the artifices of magic." Platner regarded this as a case of genuine monomania, and the propriety of his opinion was assented to by the Faculty of Leipzig.

*Case* 3. A woman had been long tortured with the most violent jealousy against one of her female servants, who, she suspected, was intriguing with her husband. She resolved to murder her, and with this view she summoned her into a private apartment, where, with the assistance of a man whom she had bribed for the purpose, she attempted to strangle her. Fortunately, the girl made her escape.

Dr. Elwert, who was charged with reporting upon this woman's state of mind, did not attempt to shew that the jealousy, of itself, could be alleged as any apology, or palliation of her offence; but he gave it as his opinion that this passion, indulged in for a length of time, had disturbed the sanity of her mind, and had induced a state of genuine monomania.

*Case* 4. A woman, who had been long the victim of religious infatuation, murdered her neighbour's child, and justified this atrocity by saying, "that death alone could save the innocent one from the sins and allurements of the world." She was regarded as a monomaniac, and confined in an asylum.

A different opinion was taken of the following case.

Peter Nielsen, a workman, on the 27th April, 1827, drowned his four youngest children, in order, as he said, to save them from the miseries of want and beggary. This man had maintained among all his acquaintances an estimable and good character, and numerous were the witnesses who appeared on his trial to testify in his favour. For some time preceding the awful deed, his affairs had become much embarrassed, and the prospect of his family being reduced to beggary had driven his mind to despair. In this state of mental wretchedness, the thought of murdering his little ones seems to have occurred to him, not once only, but on different occasions, as he had been heard to say—"il faut soustraire les pauvres enfans au malheur qui les menace, en leur donnant la mort, qui est mille fois préferable." Immediately after the perpetration of the deed, Nielsen went to denounce himself before the magistrates, expressing no regret at what he had done. He was tried, and condemned to execution; but this sentence was afterwards commuted for that of imprisonment for life.

We are of opinion that this last case was one of as genuine insanity as any of the preceding. The sanity of the poor man's mind had unquestionably been deranged by the exaggerated apprehensions for the future welfare of his children. We are well aware of the pernicious consequences to which a doctrine founded on these views may occasionally lead, and we know that some writers have attempted to establish the very dangerous maxim, that all violent passions induce a temporary madness, during the excitement of which the individual cannot be regarded as a responsible agent. If, indeed, any strong emotion or passion has occupied the mind for a *great length of time*, it is quite possible that the intellect may become perverted or deranged (as was alleged by Elwert, in one of the cases cited above); but that any sudden paroxysm of feeling can be admitted as an apology for a criminal act, is opposed by every maxim alike of morality and of political justice.

M. Henke, one of the highest authorities on these matters in Germany, very distinctly declares, "that the strong passions may, indeed, abolish the moral liberty of the person, but that this circumstance, however, does not exonerate the criminal. Man is a being endowed with a reason to point out to him his duties and to regulate and control his passions; and if he wilfully permits that reason to be blinded, it is but fair that he must abide the consequences of his own conduct." M. Henke admits that certain temperaments and states of the system, more especially the bilious and hypochondriac, predispose an individual to fits of extravagant passion, but he rejects all apologies for crime drawn from such considerations, and admits only *positive* disease as a means of justification.

M. Wildberg has expressed similar sentiments. "The medical jurist," says he, "would sacrifice his honour and conscience, if he attempted to excuse a crime by assimilating the delirium of passions, which have subjugated the reason only because they have not been kept within bounds, to a positive derangement of the mind. Those, who are accustomed to observe mankind, will readily acknowledge that habits of irreligion and of giving way to excesses of passion are quite sufficient to lead on to the most dreadful outrages men, who are in perfect possession of their reason."

M. Feuerbach expresses himself thus: "A criminal action, committed in a state of mind which precludes the moral liberty of the individual, ought not to be the less punishable, if the cause which has occasioned this state can be charged to the wilfulness of the offender."

The following propositions embody some of the most important medico-legal doctrines entertained at present in Germany:—

1. There is one form or species of monomania, which primarily springs from and consists in a disease or irregularity of the affective powers, and of the will (monomanie instinctive.)

2. It is still a question of dispute whether the intellect or reason is *necessarily* and *invariably* deranged during the paroxysms of instinctive monomania.

3. Among the accessory signs of instinctive monomania it is proper to attend especially to the corporeal or physical phenomena, which indicate the existence of, or disposition to hypochondriasis, epilepsy, hysteria, and to chronic, abdominal, and cerebral disease.

The puerperal state also, and the changes which the system undergoes at the period of puberty, have a marked influence in predisposing to this species of insanity. The character and habits of the person, the nature of the offence, he may have committed, and the conduct he displays afterwards, should all be duly weighed in our diagnosis of instinctive monomania.

4. The other form of monomania, the "monomanie raisonnante," consists primarily in a delusion or hallucination of the reason, leading often however to an extravagant and incontrollable state of some of the moral powers or emotions. This form of monomania is frequently the precursor of a general insanity affecting all the energies of the mind, intellectual as well as affective.

5. Violent and excessive passions may certainly overwhelm and, as it were, lock up the reasoning faculties for a time; but this temporary insanity must not be assimilated to monomania, which is truly and strictly a diseased state of the mind, beyond the control of the person affected.

6. It is to be remembered however that a genuine monomania may be actually induced by the long-continued or extravagant indulgence of guilty passions. When once the disease is established, the person cannot justly be considered responsible for his actions, however blameworthy he may have been previously in giving way to them.

7. There is a state of mind, characterised by a sort of exaltation or excitement induced partly by unchecked and violent passion, and partly by an incipient insanity. All medical jurists however do not agree in recognizing this condition of mental infirmity.

8. We must not confound with passions which are under the dominion of the reason, certain states of unnatural desire depending upon a real disease of the nervous system, and inducing mental alienation. There are for example certain neuroses of the genital organs, the paroxysms of which diseases are usually complicated with complete mental derangement. Such cases however are very rare, and M. Henke very properly inculcates the necessity of a most conscientious and upright scrupulosity in examining all alleged cases of this nature.

9. It is still a matter of dispute whether in partial insanity the patient ought to be considered responsible for offences, which seem to be quite unconnected with the objects of his delusion.—*Annales d'Hygiene et de Medecine Legale.*

---

Remarks on the State of Medical Doctrine in France—Endermic Treatment of Diseases—On Mercurial Tremor.

The treatment of diseases by the endermic method, at least by that form of it which consists in the application of medicines to the surface of the denuded cutis, has been much more extensively employed, and according to published reports, has been much more decidedly useful in France than in this country. Whether this difference is chiefly to be attributed to the greater vivacity of imagination, and love for every novelty of medical, as well as of modish fashion, which ever has, and ever will characterise our light-hearted neighbours; or whether their diseases are on the whole less violent, and therefore more easily curable (and this consideration by the bye is too often neglected in making comparisons between domestic and foreign therapeutics); or lastly, whether we Englishman are so perversely attached to all "established institutions," that we dread any innovations, even of truth, until they are fairly forced upon us, we shall not presume to determine. Probably each of these causes has had its influence. Certain it is, while the French journals teem with reports of cases of neuralgia, and other affections of the nerves, of intermittent fevers, of diseases of the heart, &c. &c. successfully treated by the endermic method, the English ones exhibit only about once in a quarter or six months, an example or two of amaurosis, or of tic douloureux, in which the application of strychnine, or of morphia has been tried, and this too very generally with only partial benefit. We do not hesitate to confess, that our experience of French, and we may add, of other foreign, journalism does not lead us to place very great reliance on the therapeutic precepts of the continental physicians. Their chief excellence unquestionably is rather as pathological observers, than as judicious practitioners; and their excessive devotion of late years to the mere anatomy, as it were, of diseases has, we much fear, not unfrequently led them into many extravagant errors. In almost every number of this Journal, our readers will have observed that, although far from undervaluing or disparaging the study of the morbid characters discoverable on dissection, we have invariably deprecated that medical materialism, which has misled so many in their researches as to the true nature, and consequently as to the successful treatment of diseases. Numerous are the diseases which cannot be detected by the scalpel, or by the microscope; and to arrive at any thing like a rational understanding of their correct etiology, we must invoke the aid of other means of enquiry, such as the minute study of symptoms, and of the operation of our remedies, and the observance, and when it is possible, the chemical examination also of the excretions.

In the French capital at the present day, there are three dominant sects, or schools of physicians; the Anatomical, or physiological; the Hippocratic, or rational; and the Eclectic, which forms what may be called a juste milieu between the two former. The chief supporters of the anatomical school are Broussais, Bouillaud and their immediate followers, comprising a large proportion of the younger physicians in Paris, and the majority of the medical men in the provinces, and also, we should think, in their army and navy. We are not much surprised at this. There is certainly something very seducing in the authoritative and dogmatical enunciation of the Broussaian doctrines. They are readily intelligible to the student of medicine, provided only he is willing and ready to yield implicit credence to whatever is told him in books and lectures, and when once admitted, they save him a vast deal of patient enquiry, and give him at the same time a most unhesitating reliance in his own practical skill. The scalpel and the lancet may be said to be the armorial bearings of the anatomical physician; with the one he discovers the nature of the disease; with the other he effects a cure of it.

The doctrines, and the data on which these doctrines are founded, have, we are told, all the demonstrative characters, and all the unerring uniformity of physical laws and of physical phenomena. Medicine has in their hands assumed many of the attributes of the exact sciences; and in truth the results of some of the anatomists' practice appear to be so certain—either speedy cure or rapid death—that they do not seem to have much overshot the mark in their claims and assertions. The anatomical physicians may be said to constitute the sect of the modern dogmatists. Like their brethren of antiquity, they assume the truth of certain doctrines, and their practice is guided not so much by the existing symptoms of diseases, as by their presumed and alleged organic causes. They are the genuine Whigs of medical literature. Opposed to almost all the reasonings and experience of bye-gone times, they love and follow whatever is striking and new: they have perceived some of the errors of their forefathers, and not satisfied with renouncing these, they have determined to abandon even the very high-road, to which these errors were as lanes and alleys; they have constructed theories which, they wish us to believe, are beautifully simple, and comprehensive, and which, may be worthily likened to the captivating doctrines of purity of election, and of universal suffrage so loudly recommended by their brethren in politics: they are averse to all moderate measures of treatment, scouting them as mere bit-by-bit reforms, and they prefer bold, decided, and even violent remedies, without much regard to the ultimate consequences.

Upon reading long records of cases in some of the most recent French works, and these too of established reputation, the attentive reader cannot fail to remark that few, if any, patients are recorded as incurable, or as having been discharged from the hospital unrelieved. They all most surely either recover perfectly or they shake off their mortal coil; and thus in either case, there is at least an obvious termination. Now we do not hesitate to affirm that judging from these very reports, an impression has been strongly left upon our minds, that the exit of many a patient has been accelerated by the heroic practice of these anatomical physicians. We have suspected this to be the case especially in those cases where any disease of the heart has been present. Every practical man knows, or ought to know, that a violent operation of any sort may prove almost immediately fatal, when the heart is organically diseased. We have known a drastic purgative have this effect more than once; and indeed any impression which induces syncope, or a tendency to that state is most dangerous.

Now there is scarcely one form of cardiac disease, in which bleeding, and that too very generally by large and repeated venæsection, is not recommended and used by Bouillaud, for example. Fortunately the English physicians have avoided this injudicious practice; and almost all our own recent authors have pointed out the superior advantages of local over general depletion in chronic affections of the heart. Another feature of the reports to which we are at present alluding, is the extreme paucity of merely functional disorders which they exhibit.—We rarely read of any of the class "neuroses" occurring in the wards of the La Charité, or of the Val de Grace hospitals. Asthma, chorea, and hysteria are seldom mentioned by name; and amenorrhœa, chlorosis, &c. seem to be equally uncommon.

Indeed with the exception of bronchitis, pleurisy, pneumonia, diseases of the heart, gastritis, gastro-enteritis, arthritis, and one or two others, the whole nosological catalogue might be expunged; and then with regard to the materia medica, we might, after reserving the lancet, the cupping instruments, leeches, croton oil, and a simple ptisan or two, vote it as utterly useless.

To such an extravagant extent has the depletory practice been carried, that an express "formula" for venæsection has of late years been laid down for the treatment of some diseases. We were lately reading a clinical lecture by Professor Bouillaud on acute rheumatism, wherein we are recommended to bleed patients suffering from this malady twice on the first, and also on the second day after admission (provided the symptoms be decidedly inflammatory), and then for several days successively to repeat without fail the operation, at least, once. This is the "saignée formulée" of the energetic physician of the La Charité. Colchicum appears to be rarely used, and the doses in which it is exhibited are usually very small. The preparations of mercury are seldom even alluded to; and we do not recollect at the present moment of reading that this most potent of all anti-rheumatic remedies was pushed to the extent of inducing salivation, in a single case out of fifty and upwards, which have been detailed, within the last twelve months, in the pages of the Journal Hebdomadaire, the legitimate oracle of the doctrines of Broussais, since his own Journal became defunct about two years ago. So much for the sect of Anatomical physicians of the present time. The sect arose and has flourished most in France. It has done very considerable benefit to medical science, by correcting many errors of theory, and by simplifying the practice in various diseases; but it has been far too ambitious and exclusive to be truly useful. Most of its members are believers in the doctrines of phrenology; and it will be observed, that the materialism (we do not involve any offensive meaning in this word) of these doctrines is allied to and harmonizes with the fundamental principles of the so-called physiological medicine.*

Directly opposed to the anatomical physicians, is the sect of the modern Hippocratists, or rational empirics. The leading advocates of this sect in Paris are Recamier, Bayle, Cayol, Gibert and Martinet; and the periodical which is devoted to their cause is the "Revue Medicale, ou Journal des Progrés de la Medecine Hippocratique."

These gentlemen profess to follow, not so much the doctrines and practice, as the discipline and principles of investigation inculcated by the Coan sage. Although they are not so entirely imbued with a love of his opinions, that they subscribe to the existence of four primary elements,—air, water, fire and earth, of which the body is according to him composed, and from which are derived the four primary temperaments,—the dry, humid, hot and cold—they recognize and admit, at least to a certain extent, the correctness of that hypothesis, which ascribes all the movements and actions of living bodies to a peculiar principle, denominated by Hippocrates "nature" (Φυσις) —the superintending power which influences all parts of living bodies, directing their functions in health, and exciting certain salutiferous processes for the removal, and counteraction of all morbid operations. Hippocrates attributed indeed a kind of intelligence, or power of active discrimination to this principle, which prompted it, as it were, to favor and encourage all actions which

* We observe that Broussais has within the last two months commenced an extended course of lectures on phrenology in Paris. The Lancet has promised to introduce them to the English medical public. We hope that our active cotemporary will speedily fulfil his promise. The Journal de la Societé Phrenologique comes regularly under our notice, but unfortunately there is never any communication in it, worthy of our pages.—*Rev.*

are beneficial to health, and to oppose and neutralise whatever has a tendency to induce disease. For example, whenever the stomach is suffering from over-repletion, or from the presence of any noxious substance, forthwith the Φυσις stimulates the organ to reject by vomiting the offending matter. The phenomena of a hot burning fever afford another illustration: in it all the energies of the body appear to be preternaturally excited; Nature therefore with the view of counteracting this morbid state of excitement, impairs the powers of the stomach, and the mouth and fauces become parched—the thirst, and anorexia therefore may be regarded as salutary means, instituted by this presiding principle of Nature, to restrain and subdue the feverish agitation of the whole system.

The most important duty of the physician in the treatment of disease, inculcated by Hippocrates, and observed by his followers of the present day, is to watch the operations of this nature, with the view of promoting those actions which appear to be salutary, and of checking, or suppressing those which appear to be hurtful. According to this doctrine, the chief study of a medical man should be semiology, or the careful observance of all the existing signs, or symptoms of a disease, and he should avoid having his mind perplexed with etiological speculations as to its supposed seat and essential nature. The tendency of such precepts is to induce great caution in the treatment of diseases;—much is left to the superintending influence of Nature; and violent and very energetic remedies are not generally resorted to, unless the symptoms obviously denote a strongly hypersthenic excitement. The "medecine expectante" is characteristic of the Hippocratists, and is antipodal to the "medecine heroique ou abattante" of the Anatomical physicians. These latter we styled the Whigs of medicine; and well do the Hippocratists deserve to be called the "good old Tories." Their inviolable attachment to antiquity, their love for whatever is the established order of things, their dread of innovations, their opposition to all changes, and even to many improvements in medical science (such as the use of auscultation), their preference of mild to violent remedies, and their implicit credence in the salutary and self-correcting operations of the system itself, well entitle them to this appellation. It is worthy of notice that most of the medical Tories in France (and we might add, in England) are at the same time political Tories. Dr. Cayol, for one, was deprived of his professional chair, at the accession of Louis Philippe, in consequence of his attachment to the cause of Charles X.

The other leading pathological doctrine of Hippocrates, that, which regards the fluids as the primary seat of almost all diseases, has not received, we think, from the physicians who appropriate to themselves the appellation of modern Hippocratists, the attention which it, to a certain extent, merits. Of late years the doctrine of a limited and partial humorism has been gradually, and steadily advancing into favor with the best observers of disease; and we must confess that by far the most valuable part of our information on this subject has of late years been communicated not by the modern Hippocratists, but rather by the sect of physicians, which remains to be noticed, we allude to modern Eclectics. Among these are many, perhaps most, of the best physicians in Paris, as Andral, Chomel, Louis, Bricheteau, Marc, &c.

Far from being indifferent to the study of morbid anatomy, it is well known that the eclectic school ranks among its disciples some excellent morbid anatomists. Indeed M. Andral is acknowledged by the profession to be the first pathologist in the world, and M. M. Chomel and Louis have already earned a reputation only second to his.

Yet with all their minute knowledge of the morbid lesions in almost every disease, and their unqualified estimation of the paramount value of such knowledge, the Eclectics have never permitted themselves to become such medical materialists as to believe that any physiological action, healthy or diseased, can be well explained or understood by reference solely to the normal, or abnormal physical state of any *one* organ. They have justly inculcated the necessity of minutely exploring the condition of all the leading functions of the body in disease, and more particularly in those maladies, which at their very outset affect the whole frame—we allude to the multiform family of fevers. Instead of localizing the primary seat of fevers in the brain, or in the lungs, or in the heart and bloodvessels, or in the stomach and intestines, they have wisely taught us that all these organs are, or at least may be, involved in the morbid action simultaneously, and may continue to suffer together throughout the whole course of the disease; and, moreover, that any mode of treatment which is exclusive, and directed to the relief of but one organ or one set of organs, must be, as a general rule, unwise and hurtful. They have extended their researches to the examination of the fluids, as well as of the solids of the body in disease, and they have thus laid the foundation for that rational Humorism which has sprung up of late years. They have diligently watched the symptoms which are attributable to a disturbance of the functions of the nervous system, and pointed out the influence which such disturbance exercises in modifying the features of many of those diseases, which, by the anatomical physicians, are affirmed to be mere inflammatory affections of certain organs. Take, for example, that most obstinate malady, hooping-cough. With many, it is a favourite doctrine that hooping-cough is essentially a bronchitic affection, and that, therefore, the antiphlogistic treatment is invariably and necessarily the only safe means of cure. The unprejudiced physician, however, will soon discover that the inflammation of the air-passages (if it be present at all) is always accompanied and modified by a peculiar disturbance of the functions of the nervi vagi, and this very disturbance constitutes the real and proximate cause of the disease, the bronchitic affection being, in fact, a merely contingent and occasional adjunct, or (as the French will call it) "epiphenomenon." We do not wish to undervalue the importance of regulated depletory measures in the early stage of pertussis; they are generally necessary and always useful, but nevertheless they will seldom or never cure the disease. There seems to be a peculiar morbid sympathy between the branches of the nervi vagi which are dispersed on the stomach, and those which supply the lungs, and the successful treatment of this disease often depends upon regulating and controuling this sympathy. Hence the utility of antacids, antispasmodics, and tonics, in the later stages of hooping-cough.

We need not adduce other examples to prove how necessary it is not to limit our views of disease within certain bounds of our own making, nor to attempt to simplify too much what Nature has made complex. The Eclectic physicians shew their wisdom by the more liberal and comprehensive views which they have taken. While they have not rejected the introduction of any new methods of examining disease, or of any new methods of treating it, they have faithfully retained all the good parts of the practice of the olden times, and eschewed only the obsolete modes and maxims of our forefathers. Following the example of Brother Martin, in Swift's Tale of the Tub, they have renounced the extravagances of the Lord Peters of medicine, but have most fortunately, at the same time, avoided the errors of the opposite extreme, and not indulged in all the incredulous scepticism and presumptuous arrogance of the Presbyter Jacks. They are, in short, the conservative Whigs or liberal Tories of modern physic, willing to encourage all rational reforms and substantial improvements, but steadfastly opposed to all extravagant and Utopian notions of uniform and universal equality.

We have been gradually led on to indulge in the preceding random observations on the different sects or schools of medical practice prevalent at the present time in France, after having, for several successive days, been poring over the wearisome pages of the Encyclographie des Sciences Medicales; which, as many of our readers may know, consists of a reprint of almost all the medical periodicals published in Paris.

But we must now return to the point whence we started; we mean the consideration of the endermic method of treating diseases. M. Raciborski has communicated several papers to the Journ. Hebdom. on this subject, and we propose to extract such of his observations as may seem worthy of notice.

### Endermic Use of Quinine in Agues.

The endermic use of quinine, in cases of intermittent fevers, has been adopted by several of the best physicians in Paris. It is well known that, in many agues, the mucous membrane, not only of the stomach, but also of the large intestines, is in a state of congestion or of actual inflammation. The exhibition of quinine or of any other tonic by the mouth, or in the way of enema, must, under such circumstances, be positively injurious, and it has been with the view of avoiding any aggravation of the intestinal distress, and yet overcoming the peculiar and unknown state of the system on which the periodic recurrence of a morbid paroxysm, in intermittent diseases, depends, and which is so generally controlled and removed by bark in all its forms, that the endermic use of quinine has been so strenuously recommended, especially by those physicians whose minds are ever haunted with the dread of gastrite or gastro-enterite.

Five cases, treated by M. Chomel at the Hôtel Dieu, are reported by M. Raciborski.

The first was certainly a very mild case. A man 28 years of age, robust, and hitherto in perfect health, was seized with a febrile paroxysm which lasted for four or five hours, after exposure to cold and damp. The paroxysm returned regularly every second day, and on the intermediate days the patient was quite apyrectic. On the morning when the sixth paroxysm was expected, eight grains of sulphate of quinine were applied on the epigastrium, which had been previously denuded of its cuticle by strong ammoniacal pommade. The paroxysm was quite checked, and, although the patient remained in the hospital for another week, there was no re-appearance of any agueish symptoms.

The second case occurred in a middle-aged man, who had suffered from tertian fever in the preceding Autumn, and in whom the recurrence of the disease at the period of his admission seemed to have been induced by a violent mental emotion. He had already experienced eight paroxysms within the last 16 days. When admitted, he was quite free from any feverish disturbance; a light demulcent diet was ordered. Two paroxysms occurred in the hospital. Two grains, therefore, of quinine were ordered to be applied on a blistered surface of the epigastrium. On the following day, the paroxysm returned, but at a later period of the day than hitherto, and it lasted only one hour, instead of four or five, as on former attacks.

The application of the quinine was repeated daily, and all disposition to any recurrence of the intermittent illness ceased entirely.

The third case was similar. Only four paroxysms had occurred before the endermic use of the quinine was tried. The application of four grains daily effected a complete cure.

The fifth case, one of tertian fever, like all the preceding examples, was more severe; the disease had been of longer duration; the patient had suffered from ague before; and moreover the spleen was felt to be perceptibly enlarged. Six grains of quinine applied to a small denuded surface on the epigastrium an hour before an expected paroxysm, caused it to be much less severe and less prolonged than any preceding one; and by repeating the application once or twice again, there was no recurrence of any feverish attack.

[Our readers will probably agree with us that the preceding cases do not afford any very satisfactory data for the endermic treatment of intermittent fever. We give them as we find them reported in our Continental cotemporary. The doctrine of those metaphysicians, who teach us that the relation of cause and effect is nothing more than that of mere antecedence and succession, appears to influence most of the medical reasonings of our lively neighbours. A mild case of ague occurs; the patient is admitted into a hospital; some quinine is applied once or twice to a blistered surface; the disease becomes milder, and is cured:—therefore, the cure must be attributable to this item of the treatment.—Q. E. D.—*Rev.*]

### Endermic Use of Digitalis in Organic Affections of the Heart—Mercurial Tremor.

M. Raciborski reports four cases of heart disease, in which the endermic use of digitalis was, we are told, productive of beneficial effects.

The first was that of a Polish woman, 44 years of age. The rational and auscultatory symptoms indicated a general hypertrophy of the heart, and induration of the valves; and, in addition to this disease, there was a considerable effusion into the abdominal cavity. The urine was very scanty. A potion, containing 10 grains of powdered digitalis, was ordered to be taken in divided portions, and this dose was repeated for the following two or three days. In consequence, however, of extreme nausea being induced, the powdered digitalis was applied to a blistered surface on the epigastrium. The peculiar sedative influence of the medicine continued, we are told, as if its internal use had been persevered in. [Surely this is a very insufficient proof of the efficacy of the remedy.—*Rev.*] After the lapse of a fortnight, the blister having already healed, a mixture, containing 10 grains of the digitalis, was again ordered for internal administration. The dose was raised to 15 grains. The poor patient became worse. On the second day preceding her death, we are informed that she suffered much from dyspnœa, vomiting, headache, and sleeplessness. The palpitations of the heart were very feeble; the pulse was 57. She was bled to six ounces (!) The blood drawn presented a large quantity of serum, and no trace of buffy coat on the coagulum (!!) The distressing vomiting (induced, no doubt, by the poisonous influence of the foxglove) continued to the last.

[If we were desired to give a name to the preceding case, we should have no hesitation in naming it—fatal effects of an overdose of digitalis, in a patient labouring under organic disease of the heart. We could not find a more apt illustration than what this case affords, of some remarks on the dangerous practice of the anatomical physicians, in a former part of this paper.—*Rev.*]

The following case is certainly more pertinent than the preceding one. A woman, 28 years of age, had been in the Hôpital de la Charité for rheumatism and palpitations of the heart, in Oct. 1833. She was cured by the internal and external use of digitalis in large doses. In the following March, the heart symptoms returned, and the patient having, on the former occasion, suffered distressing sickness from the internal use of the remedy, requested that the external use alone might be tried. The palpitations of the heart were strong, and one of the bruits was accompanied with a very distinct blowing or whizzing sound. On the 6th of March, the pulse being 100, 12 grains of powdered digitalis were sprinkled on a blistered surface of the epigastrium. On the following two days, the pulse had fallen to 82, and the palpitations had been decidedly less frequent and less violent. The pulse gradually fell to 64, and all the symptoms became so much mitigated that she left the hospital. It is not stated in the report, how often the application of the digitalis was renewed; but we are led by the context to suppose, that it was every second or third day.

The third case affords another most distressing example of the fatal effects of *doing too much* in extensive and serious disease of the heart. The patient died *in consequence* (M. Bouillaud would no doubt substitute "*in spite of*") of bleeding and large doses of digitalis. Truly, Sangrado and his worthy pupil could not possibly have despatched their patients in a more summary manner.

From these melancholy details, we pass to notice a few cases which are adduced to prove that the muriate of morphia used endermically affords great relief in many nervous disorders. A rather curious case of a young man, who had suffered from vomiting every night (at least whenever he took food soon before going to bed), for a period of six weeks, is detailed. This unpleasant symptom was removed by blistering his epigastrium, and applying to the raw surface a grain of the muriate of morphia, for five days successively.

No internal medicines were used. It is quite possible that a single dose of opiate, taken inwardly, would have been as, or perhaps more speedily successful. Two other cases are given, in which the vomiting had been even still more obstinate, had resisted internal remedies, but where it yielded to the endermic use of the acetate of morphia, in doses of from one-half to a whole grain applied for several days in succession.

The following case of MERCURIAL TREMOR successfully treated in a similar manner is instructive. A man, 34 years of age, who dealt in mercury, was admitted into La Charité Hospital on May 1834. Three months prior to his admission, he began to be affected with tremblings of the upper extremities; and soon afterwards the lower limbs were similarly affected. These tremblings had gradually increased so much, that he could with difficulty hold a cup in his hand, or direct his food to his mouth. When he attempted to stand, his limbs bent under him, and he must have fallen, if not supported. There was no headache. His pulse was natural, rather slow.

A long narrow blister, nearly a foot in length, was applied over the spinal column, and when the epidermis was separated, a grain of acetate of morphia was sprinkled and retained on the denuded surface. In the course of five days, a decided amendment was observable. Enemata of musk and camphor had been administered occasionally. The same treatment was persevered in for a week more, and a complete cure was obtained.

With respect to this sort of shaking palsy, the "tremblement mercuriel," of the French, it is not unfrequently observed in workmen who are exposed in their trade to the fumes of, or to the mere contact of quicksilver. Hence miners, gilders and barometer makers, are those in whom the disease is usually noticed. The disease usually creeps on slowly and gradually. At first the person finds that his arms and hands are unsteady and trembling, and that he cannot keep them quiet for any length of time. Then his limbs become week and tottering, whenever he attempts to walk: his speech also becomes affected, and sometimes even the effort of chewing is accompanied with a convulsive agitation of the muscles of the cheek and throat. If after the occurrence of these symptoms, the patient is still exposed to the noxious influence of the quicksilver, loss of memory, sleeplessness, delirium and death may ensue; but this is necessarily of very rare occurrence, as he is generally quite incapacitated in the earlier stage of the disease from following his work. The entire surface of the body becomes in some cases of a brown colour; the pulse is usually slow; the bowels are not much affected, except with flatulence.

In this form of quicksilver-poisoning, the salivary glands are but little, if at all, affected. Death has been in most of the fatal cases owing to marasmus and a general sinking of all the energies, especially those immediately dependent on the nervous system. It appears from two or three cases on record that a single exposure to the fumes of mercury has been sufficient to induce the disease.

Dr. Christison mentions a case, in which a barometer-maker and one of his workmen, who were accidentally exposed one night during sleep to the vapours of mercury from a pot on a heated stove, were most severely affected, the latter with salivation which caused the loss of all his teeth, and the former with shaking palsy, which lasted his whole life. Generally however, the disease comes on very gradually, and only after repeated and lengthened exposures to the morbific cause. Dr. Darwall describes the case of a man, 62

years of age, in whom the disease had existed for twenty-five years. " At the time we saw him," says he, "all the voluntary muscles were violently agitated, so that every attempt to speak was interrupted by the spasmodic contractions of the jaws and tongue. His head was in a continual see-saw; he could not hold any thing in his hands. Any attempt to obtain control over his muscles invariably rendered the convulsions more violent. Mental agitation increased them. His teeth had been lost for many years; they had fallen out without being decayed. His appetite was good, his sleep sound, and his evacuations were voluntary."

Many of the symptoms of mercurial tremor indicate a very marked resemblance of this disease to the chorea of young people. The agitation of the arms and hands, the shuffling dragging gait, the confusion of the speech, and the occasional imbecility of the mental powers, are common to both. Perhaps too the etiology of both diseases, were it correctly known, might point out the intimate alliance between them. Indeed a general trembling of the body, whether induced by chorea, mercury, the abuse of ardent spirits, wine, coffee, opium, &c. by excess in venery, by masturbation, or by the gradual decay of the animal powers, as old age advances, may, it seems probable, be in every case referable to a common cause, viz. some temporary or permanent affection of the brain, or of the spinal marrow.

A violent mental emotion will often produce a universal trembling of every part of the frame; and many cases of shaking palsy, occurring in prematurely old constitutions, may be traced to some paroxysm of passion, anger, grief, or even of joy.

---

### Baron Alibert on the Cheloid Tumor of the Skin.

The cutaneous affection which has been named, " keloide" or " cheloide" (from the striking resemblance which in shape it often bears to the figure of a crab) was first accurately described by Baron Alibert in his *Monographie des Dermatoses.* Some writers have described it under the appellation of " cancroide" or of " cancre-blanc." Bateman at first disputed the existence of any such disease; but subsequently he discovered his error, and within the last twenty years so many pupils have attended Alibert's lectures at the Hôpital St. Louis, that most medical men are, he says, now well acquainted with its characters. It is indicated by a hard and resisting excrescence of the skin, usually flattened in the middle and more prominent at the edges, throwing out from the sides branches or prolongations, which, when lengthened, look like the legs of a crab. These prolongations have sometimes the appearance of irregular scars from a burn. Occasionally the diseased skin exhibits several oblong, cylindrical knobs, or lumps, more or less prominent above the rest of the surface, and imbedded, as it were, in the cutaneous tissue.

The first case, which the Baron ever saw, occurred in a young female. The excrescence was situated between the mammæ, and measured about two inches and a half in length, and about one in width. It projected for a line or so above the surface, and was of a deeper red colour than the surrounding integuments. From each side there extended a bifurcated branch or prolongation, giving the cheloid patch a certain degree of resemblance to the body and legs of a crab. Independently of an almost incessant and very annoying pruritus, the patient felt at times sharp darting pains through the tumor. The case had been submitted to the examination of several eminent surgeons, and the general impression was that the disease was of a cancerous nature. M. Alibert however, perceiving in its appearance very marked difference from that of ordinary cancer of the skin, did not assent to this diagnosis, and the correctness of his opinion he soon had an opportunity of confirming by the inspection of several other similar cases. One of these occurred in the person of a distinguished actress. The tumor being situated over the sternum, the lady very sedulously concealed "cette disgrace" with a large medallion, which she wore constantly appended to her necklace. It was hard, flattened on the surface, somewhat depressed in the centre and "bombée" in the circumference, and it was of an oval or condyform shape. It felt on examination, as if it was implanted in the proper substance of the skin by four roots or prolongations, which, in allusion to the name of the disease, might be considered as the legs of the crab. Round the periphery of this "bizarre excroissance" a multitude of minute blood-vessels were seen at every point. Since the date of these cases, the Baron has examined a great many more, and in all of them the characters were nearly alike to those now described. The "cheloide" may therefore be described as a cutaneous excrescence of a square, oval, or elongated cylindrical shape, hard and resisting to the touch, streaked and traversed by numerous reddish lines on the surface, of a shining aspect, and of a deep red colour, which disappears on pressure, but returns immediately whenever the pressure is withdrawn. Baron Alibert has seen not fewer than seven or eight cheloid excrescences, on the neck of one patient. Although of different forms and sizes, they were all provided with the lateral elongations which have been compared to the legs of a crab. When the patient was quite cool, the cheloid tumors gave little or no inconvenience; but when she was heated, they were affected with a most troublesome lancinating pruritus.

When the cheloid is of a lengthened shape and deeply imbedded in the cutaneous tissue, it sometimes feels to the finger as if a worm had been inserted under the skin. The parts most frequently affected with this disease are the upper and fore part of the thorax above or between the mammæ, the neck, back, shoulders, arms and thighs. It has been seen in one or two cases on the face. This "dermatose" is sometimes quite indolent, continuing unchanged for many years; at other times it is accompanied with intolerable itching and with pricking darting pains, as if dozens of burning needles were thrust into the part. The pains sometimes extend to the adjoining tissues, and occasionally they are felt sharply in the neighbouring viscera. One patient expressed her suffering by saying that she felt as if her chest would fairly burst open; another, a lady, wrote to the Baron that "elle avait au sein l'aspic de Cleopatre;" a third, a simple country-girl, thought that a venomous toad had crept somehow into her flesh, and was consuming her vitals. The pruritus is always most annoying at night when the patient is warm in bed.

It must appear strange that the cheloid excrescence should ever have been associated with and mistaken for cancerous affections, as the characters of the two diseases are very different. Independently of the very peculiar shape of cheloid tumors, they never become ulcerated, but always remain in an indurated condition. The only feature of resemblance which cheloid bears to the groupe of cancerous dermatoses is "par les douleurs lancinantes qu'elle provoque, et par le siege qu'elle occupe sur le tegument." Cheloid cannot be regarded as a dangerous disease; for in not one instance has the Baron witnessed "aucun accident facheux," and he has known it to exist for a long series of years without occasioning much inconvenience. He tells us that he is acquainted with a young lady, whom "this indisposition" did not prevent from contracting "un marriage avantageux!" Females, it appears, are much more subject than males to cheloid. Alibert cautions his readers against mistaking the mark or scars left by scrofulous or syphilitic ulcers, or by burns, for genuine cheloid. Such scars never occasion pruritus, nor the lancinating pungent pains which usually attend the latter affection.

The existing causes of this "bizarre maladie" are very obscure. It is most frequently observed in persons of a lymphatic constitution, and when once developed, it insensibly increases in size for some time, and then often remains in a stationary condition all the rest of the patient's life. Very frequently there is no co-existent constitutional disturbance. In one or two cases Baron Alibert has seen it in the children of parents who had been affected with it.

On examining the texture of cheloid excrescences, it is found to be very compact, fibrous, crossed and interlaced, as is the case with the glandular part of the mamma, and it is almost uniformly of a white colour. It is truly a disease of the cellular tissue.*

With respect to the treatment of cheloid tumors Alibert has nothing satisfactory to communicate. In general they resist every mode of medication. Two cases only have occurred in his practice, where the tumors disappeared spontaneously, leaving however indelible wrinkles on the parts affected. A young woman had a cheloid excrescence on the sternal region. Sharp pricking pains were occasionally felt in it. From being much exposed to the vicissitudes of the weather, this patient had for several successive years repeated attacks of erysipelas, which gradually caused a complete dispersion of the diseased cutaneous engorgement. The skin of the part however remained depressed, wrinkled, and of a pearly white hue. In one case excision effected a complete cure; every part of the excrescence had been extirpated with the knife, and the arsenical paste was afterwards applied to the exposed surface. The Baron has repeatedly made trial of almost every variety of caustic remedies, such as the nitrate of silver, the potassa fusa, the muriate of antimony, also the different preparations of arsenic, mercury and iodine, but hitherto without success. Although the diseased structure may have been thereby "altered, disorganised, and reduced to a state of a suppurating sore," it was found that no sooner had the surface cicatrised, than it (the cheloid excrescence) was reproduced. The more it is irritated and tormented, the more its ramifications extend in every direction. In this point of view, cheloid has some analogy to *noli me tangere.* Sometimes indeed the disease appears to be yielding at first to the application of remedies; but almost invariably this appearance is but very short-lived, and the tumor regains its size, or assumes a larger one. It is fortunate that the disease is of little or no danger; and perhaps therefore the most advisable plan is not to interfere with it. It seldom or never ulcerates—*Journ. des Connoiss. Medicales.*

Rayer in his great work thus defines cheloid and describes its origin and progress. "It is an oval or cylindrical shaped tumor of a permanent character, reddish colour, sometimes with a smooth, but often with a wrinkled surface, and of a hard and firm consistence, without pulsation, or rushing in the interior, slightly prominent, spreading in flat patches, or sending off from the circumference prolongations which have been likened to the claws of a crab. There arises at first on some point of the healthy skin, and still more frequently on the cicatrice of a phlyzacious pustule, of a wound, burn, &c. a reddish point, of the form and dimensions of a grain of barley. It is frequently affected with severe pruritus. Several of these points have been seen arising simultaneously on different parts of the skin, which had been irritated by the development of phlyzacious pustules. I have seen them occurring on the face after confluent small-pox, and in the course of a few months acquiring all the characters of the genuine cheloid tumor." Rayer has seen five cases of cheloid: in three the tumor arose over the sternum without any assign-

* The language of the Baron we have not translated literally, and perhaps not correctly. We shall therefore give the original expressions. "La cheloide appartient, aussi bien che le genre *carcinus*, à la partie celluleuse du nevrileme. Ces deux maladies tiennent au meme groupe. C'est parce que ces tumeurs se forment dans la gaine cellulo-nerveuse du tegument, qu'elles ont une extreme propension à repulluler quand on les extirpe." Do our readers attach any definite meaning to the expressions "cellular part of the neurilema," and "cellulo-nervous sheath of the integument?"

able cause; in another, it occurred on the buttock of an adult, upon a cicatrice of an old burn, and in the last case there were two tumors, one on either cheek; these supervened after small-pox."

When the disease has existed for a length of time, its central part, or different points of its circumference, become soft, shrivelled, and wrinkly, assuming quite the appearance of a scar left by a burn. This, as well as other characters, will enable the surgeon to distinguish cheloid from scrofulous, syphilitic, or cancerous tubercles. Rayer advises that no irritant application be made to cheloid tumors. He classes the disease among the hypertrophiæ of the skin.

The following description of a cutaneous affection, given by Retz in his treatise on "Maladies de la Peau," Paris, 1790, appears to refer to cheloid.

"This disease is very extraordinary; I have only met with three cases of it. The epidermis is not affected; it is only of a deep red colour, and is raised by a collection of solid matter, which here occurs in lumps of the size of an apricot, and there in rays the length of the finger, and as thick as maccaroni; or the substance appears under the form of flattened and extensive wens, much raised, and as broad as one or both hands. These wen-like patches are singularly interlaced with bands of the same substance, of different thickness, resembling broad cicatrices, and form several folds and counterfolds, as if there were sundry cicatrices placed one on the top or by the side of another."

---

### M. St. Hilaire on Albinism and Melanism.

Albinism may be either partial, or general and extending over the whole surface of the body. In the former variety, patches only of the body are affected with decoloration, or an unnatural whiteness of hue. It is most frequently observed in the negroes, and when it occurs in them, it gives rise to a curious appearance: they seem piebald, the white patches contrasting strongly with the deep black of the rest of the surface. When the albinism is complete, we almost always find that some other tissues of the body, as well as the skin, exhibit anomalous appearances. Thus the iris is usually either colourless, or it is of a transparent red colour; and the pupil is often of an intense red, the choroid coat being then destitute of its pigmentum nigrum. This coincidence of irregularity in the dermoid and visual organs has been adduced in support of the hypothesis advanced by the German naturalists, and advocated by M. Blainville, that there is connexion between the skin and the organs of the other senses.

The constitution or temperament of Albinos is invariably lymphatic; and indeed we might, à priori, have expected that this should be the case, seeing that persons of this temperament have always a paler hue than other people. Blonde persons are always more or less lymphatic.

In consequence of the changes in the condition of the eyes, the transparency of the iris, and the deficiency or the total want of the pigmentum nigrum of the choroid, albinos cannot bear easily a strong light, and, therefore, they do not see well in open day. Their eyes are usually more or less closed, and the constant winking has led some to suppose, that the levatores palpebræ muscles have lost their accustomed power. It is well known that a similar state of the organs of vision is the natural or normal condition of certain of the lower animals; viz. of those which see best in dull light, and which seek their prey in the dusk. The powers of propagation and the mental faculties are always imperfectly developed in albinos.

Some tribes of Africans have generally regarded albinos with unkindness, and even with abhorrence, while others have superstitiously venerated them, looking upon them as superior beings, and worthy of the highest respect. The circumstance of their being, in some places, ill-treated, and often obliged to live together at a distance from the rest of the tribe, explains the error of Buffon, and some other naturalists, in supposing that the albinos form a distinct race of people. But this, indeed, is not unlike many other of the idle speculations of the ingenious Count. Albino women, although certainly not barren, are much less prolific than other females. Their offspring is sometimes quite healthy, while at other times they inherit the peculiarities of their parents. It was falsely supposed, for a length of time, that the offspring of an albino woman and of a male negro must be mottled or piebald; but this idea is quite an erroneous one, and it has been disproved by M. Geoffroy St. Hilaire, who has satisfactorily shewn, that the offspring of two persons of different normal races, as, for example, of a white and of a negro, is invariably a mule, partaking of the modified characters of both parents; while, on the contrary, the offspring of two persons almost entirely alike is variable, sometimes resembling in most respects the father—at other times the mother. This law is strictly applicable to albinos, and we see it exemplified daily in the offspring of domestic animals.

Whatever be the degree of albinism, whether it be partial or complete, feeble or intense, the person invariably retains all the distinguishing features and characters of his race.

Melanism, or that state where the natural colour of the skin is much darker than usual, is much more rare than albinism. In the human being, it has never been seen in a decided or very marked degree; but in the lower animals it is by no means unfrequent. Authors have, indeed, narrated some wonderful tales of black, or at least dark-coloured children being born of white mothers. Thus, for example, an anatomist of the sixteenth century has related the instance of a woman, who had escaped from a fire " à moitié brulée," and gave birth a short time afterwards to a child entirely black! and every one has heard of mulatto children having been born, when certainly the *married* couple have been fair, as fair could be. Whether both father and mother were so, is a different question.

Partial melanism is of not very unfrequent occurrence; but hitherto it has not been attentively studied. It is this anomaly which constitutes what have been called mother-spots, or nævi materni.* Authors have recognized two important varieties of these blemishes—1, sanguinolent spots, caused not by the deposit of colouring matter in the dermoid tissue, but by an hypertrophy of the vascular system in certain parts of the skin; and, 2, melanotic spots, occasioned by the deposit of colouring matter in the dermoid tissue.

The colour of the spots varies much in intensity, from a deep black to a yellowish-brown or a coppery red. When the spots are truly melanotic, they are indelible, and are neither effaced spontaneously, nor can they be dispersed by any remedial means. They may, therefore, be regarded, in a legal point of view, as useful adjuvants, in some cases, in establishing the identity of certain persons. They are seldom or perhaps never elevated above the surface, nor does their colour ever vary in intensity as is well known to be the case with the proper nævi materni, from the influence of external heat, mental emotions, and so forth. It is unnecessary to allude to the characters of the sanguinolent spots, or proper nævi, or to the supposed connexion between the origin of these, and, we may also add, of the melanotic spots, and certain longings, impressions, and imaginations of pregnant women.—*Gazette des Hôpitaux.*

The preceding remarks of M. Alibert are very scanty on the subject of albi-

---

* M. St. Hilaire is extremely incorrect in arranging the varieties of nævi under the general head of melanism. There is, indeed, one variety of them, the pigmentary nævi of Rayer, the " spili" of the old authors, which seems to consist in some anomalous state of the rete mucosum and of the colouring matter of the skin; but surely the other two varieties, in which either the vessels of the skin are enlarged and diseased, or there is an hypertrophied and thickened condition of the cutaneous and subcutaneous tissues, have no characters in common with ordinary melanism.—*Rev.*

nism and melanism. We shall therefore add a few particulars. The hair of albinos is usually smooth, silky, and straight, but sometimes crisped, as in negroes ; its colour is like that of cotton, or blanched silk, and very different from the golden yellow colour of light hair. The eyebrows, the beard, and the hair of the pubes are equally blanched. Albinos are generally weakly in frame and feeble in mind. Partial albinism is sometimes congenital ; at other times it occurs during the progress of life. Dr. Rayer alludes to the case of a young girl, who had spots of lentigo on the face, and leucopathic patches on the neck and body. Such patches are more frequently observed on the genital organs, and in their neighbourhood, than in other parts of the body.

Accidental partial albinism or leucopathia is most frequently the consequence of some strong mental emotion. Rayer had lately a case, at the Hôpital de la Charité, of a man 30 years of age, who, upon the loss of some property, became affected with this appearance; there were numerous white milky spots disseminated over the body and limbs, and there was a partial blanching of the hair, whiskers, and eyelashes.

General or universal albinism has been observed most frequently among the African negroes; occasionally, also, in the natives of Darien, of Brazil, Sumatra, and New Guinea, and even in Europe among whites, in Denmark, England, France, Switzerland, &c.

With respect to melanism or nigrities, we find the following remarks in Rayer's great work.

General congenital nigrities has, perhaps, never been seen ; but there are several well-attested cases of accidental nigrities on record. Thus, M. Chomel alludes to the case of an apathetic old soldier, whose skin, without any appreciable cause, became as black in some parts as that of a negro, and of a yellowish-brown in others. M. Rostan has published a case of a woman, 70 years of age, who became as black as a negress in a single night, after great mental agitation.

Partial nigrities is of not unfrequent occurrence. It is most commonly observed on the genital organs. The scrotum and penis of adults is not unfrequently of a very dark colour; and Haller has seen the same appearance in the pubic region of a female.

During pregnancy, patches of nigrities are often observed on the abdomen, around the nipples, and sometimes also on the face. Rayer has seen several cases in which the tongue became partially black, the colouring matter, of a blueish-black hue, being generally deposited on the edges of the organ, in small close spots, whence it gradually extended over its upper surface.

---

### ODORIFEROUS EXHALATION FROM THE SKIN.

There was a case lately in the wards of La Charité, under the care of M. Rayer, of this pathological curiosity. A woman, 38 years of age, was admitted in a state of extreme marasmus, and labouring under chronic peritonitis. During the eight days preceding her death, the surface of the arms and of the trunk exhaled a very strong odour of musk.

Strict enquiries were made to ascertain whether any musk, or other similar perfume, had been introduced into the wards by the nurses or friends of the patient, "et tout le monde, ainsi que la malade elle-même, nous a repondu par la negative." The treatment of this woman had consisted in the use of antiphlogistic, emollient, and revulsive remedies, such as leeches to the epigastrium, blisters and sinapisms to the legs, and demulcent drinks.

The post-mortem examination did not very satisfactorily account for the disease which proved fatal. The only lesions found in the abdominal viscera were some old adhesions of the rectum to the posterior surface of the uterus, and of the colon to the liver ; the ovaria and kidneys exhibited traces of inflammation ; the intestines were at different points congested with blood, but did not present any well-marked or very serious lesion.

These alterations do not certainly account for the extreme tenderness of the abdomen during life, for the very obstinate diarrhœa, the excessive exhaustion, and tendency to coma.

In a late number of the Annali Universali di Medicina, Dr. Speranza has reported a case, somewhat analogous to the preceding one: but in his patient the extent of surface, which gave out the odoriferous effluvia, was much more limited; for it was confined to one of the fore-arms.

He has collected the particulars of most of the cases which have been recorded; and among these, we observe that there are three, in which a strong musky smell was emitted from the armpits.—*Lancette Française.*

---

### Case of Blackening of the Skin—the Pannus Melaneus of Alibert—the Melasma of Rayer.

A woman 41 years of age, unmarried, and who had led an active and laborious life as a servant, was admitted into the Hôpital St. Louis under the care of Baron Alibert. She stated that she had enjoyed good health until her thirty-fourth year, at which time the catamenia, which had previously been regular and abundant, became very scanty, and her general health was a good deal disturbed. The complexion of her skin had hitherto been quite fair; but now it became considerably darker, and by her thirty-ninth year the brown hue had increased so much that it "attirait l'attention de tout le monde."—The skin had become also very rough and furfuraceous, and she was annoyed with a most intolerable itching over almost every part of her body. In course of time the scalp became affected with a dartrous eruption, but this gradually abated.

When first examined by Baron Alibert, this patient's general health was pretty good; her speech however was difficult like that of a person affected with partial paralysis. The colour of the skin over the whole extent of the body was nearly that of a withered leaf, darker than in any case of elephantiasis, and approaching almost to the hue of the negro. Even the lips were blackish, and the tongue which was of a pale brown colour exhibited four or five very black spots of from three to four lines in diameter. The hands were quite as dark as in a negro. The fore-arms exhibited several patches of a black colour: these patches had been the seat of an intolerable itchiness, and had become furfuraceous, before they acquired their dark hue. In no other part of the body were these dark patches observed.

This discoloration of the skin has been called "pannus melaneus" by Baron Alibert. It belongs to the class "dermatose dyschromateuse."—*Journal des Connoiss. Medicales.*

The disease in the preceding case is the pityriasis nigra of Willan and other authors. It is not a simple discoloration of the skin, for there is always more or less accompanying furfuraceous desquamation. By this compound character, the genuine melasma (this is the name given to it by Rayer) may be distinguished from partial nigrities, described in a preceding article. Dr. Willan observed several cases of this affection in children who had been born in the East Indies and brought over to England. It began with an eruption of minute papulæ, and ended with slight furfuraceous desquamation and blackish discoloration of the skin. A melasmatic affection of the surface is not unfrequent in the pellagra of Italy, and it was observed in some cases of that anomalous epidemic (acrodynia) which prevailed in Paris in 1828.

The following very curious example is related by Rayer.

A man 33 years of age was admitted into the Hôpital St. Antoine on the 27th September, 1828. His complexion was brown, and his hair was black. He was suffering with the symptoms of the epidemic. On the day of his entrance, all were struck on uncovering the patient with the black hue of the skin of the belly and trunk; the patient seemed equally astonished himself, and assured us that the skin, though always naturally brown, was very different from what he had ever observed before. This hue continued. On the 30th of September, no morbid discolouration had taken place on the face; the skin of the neck, and of the anterior and posterior parts of the thorax was brown and smooth like that of a mulatto. The anterior part of the abdomen presented a very peculiar appearance: the epidermis was detached in small laminæ about the size of a silver threepence; these laminæ were positively black both on their internal and external surface, but they appeared blacker on the skin than after they were detached. On the parts from which the cuticular lamellæ were ready to be detached, the skin was already provided with a new epidermis, not nearly so black as the preceding one, and almost of the same colour as the skin of a mulatto. The skin of the scrotum and of the upper parts of the thigh was as black as that of a Negro, but it was not smooth and shining in its appearance; desquamation was in fact going on in several places from its surface. After the coloured epidermis had fallen off, the skin assumed a brown hue."

---

### NEW MEDICAL JOURNAL IN INDIA.

We were not till late in this quarter, made aware of the existence of a new labourer in the vineyard of periodical medicine, on the banks of the Ganges. The "INDIA JOURNAL OF MEDICAL SCIENCE" completed its second volume on the 1st December last. It is now edited by Mr. Corbyn, whose name is known to his European brethren, as an early writer on the Indian Cholera. The first volume, however, was edited by Messrs. Grant and Pearson, who have retired. The plan of the work is rather too popular for this country; but is, perhaps, well adapted to India, where great numbers of Europeans are deeply interested in medical matters, without being able to procure medical advice.

We shall make occasional selections from the department of original communications; but at present, can only introduce one or two extracts. The following graphic portrait of our friend Mr. Liston, drawn on the banks of the Ganges, is good-humoured, and will excite a smile on the countenance of this talented surgeon, now a resident amongst us.

### *Sketches of the Edinburgh Medical Professors, No. 3.*

#### MR. LISTON.

"Dear Gentlemen,—The light and characteristic graver of your portrait-taker Scotus, has vividly recalled the images of the dear and illustrious names, which have rendered Edinburgh, what Professor Sedgwick calls it 'a Glorious City.'

As I hope Scotus will complete a Picture Gallery of 'the Professors,' I will not poach upon his domain, but will attempt, with your leave, to give a 'local habitation' to some reminiscences of the private Lecturers, some of whom are not less known to fame, than the Professors; and our first *subject* shall be ROBERT LISTON.

I could easily give his birth, parentage, and education, but as Surgeons, unlike Poets, are not born such, I will at once take him, 'with all his blushing honors, (and numerous imperfections) thick upon him,' as he lectured four years ago, in Infirmary Street. Suppose yourself one of 150 young men, seated in his small lecture-room, watching the preparations being brought in, especially that enormous Sarcocele, weighing 60 lbs., and requiring three apprentices to bring it in, one of their master's trophies. A carriage rattles up to the door, and a tall, rather heavily made man, with a considerable stoop of the shoulders, lounges in among a group of students, chatting and laughing; he makes for the arena, and mounts a *four-legged stool;* you observe his fine neck, and pale, manly, massy features, of which the finely cut firm mouth is characteristic; his bust would please a sculptor, and his friend Joseph has made an excellent likeness of him.

But Heavens, how awkward he is!

See how he scrapes the floor with one foot, while the other is cock'd up under the stool, as if to keep him on, and his lower lip is held in his teeth; how he swings from side to side, like an elephant in a cage, but something is wanting; ha! he seizes a *catheter*, (the Aaron's rod which vivifies this rock) he flourishes it right and left, like a broad-sword, his lip escapes from its restraint with a smack! and I am mistaken, if you do not receive sound, practical rules of surgery, usefully illustrated, and skilfully reduced to practice by operations, and you will leave him pleased and instructed, in spite of his uncouthness, stammering, and gyrations, on the antipodes to his head.

Mr. Liston is an enthusiast in his profession, and well fitted to inoculate his students, with his own cool, bold, decided spirit. He has been gifted by nature, with what at first would appear an imperfection in a Surgeon, the most enormous hands I ever saw on man, but like the Elephant's trunk, they are capable of the most minute, as well as the most powerful actions, and whether he breaks down a cataract, excises a hip, frees a hernia, or extracts a stone, he is a first rate operator. The success is equal to his despatch; in lithotomy the patient is usually returned to bed in 2 or 3 minutes from the commencement of the operation, and the deaths out of his numerous operations have been very rare. After lithotomy, he inserts a gum elastic tube into the bladder for some days, to prevent infiltration of urine, in the tract of the wound, which he justly considers a great source of mischief. Light dressings, and the absence of all irritation, are strenuously inculcated by him, especially where union by the first intention is looked for. I think that I hear him characterise " more suo," the proceedings of the Montpellier Surgeon, described by Mr. Atkinson, who sewed over the surface of the stump *continuously*, &c., to procure " la reunion par premiere intention!" Mr. L. despises medicine, and its professors, as much as he adores surgery, he thinks that,

One Physician like a sculler plies,
He gently paddles, and his patient dies;
But two Physicians, like a pair of oars,
Quickly waft you, to the Stygian shores.

He was wont to say, that a Physician was like a blind man with a cudgel, trying to separate two persons fighting, as likely to knock the injured man on the head, as the aggressor.

Mr. L. when off his guard, (for he can smile, and pleasingly too when he likes) is rather overbearing and dictatorial, failings which are somewhat cherished, by the subserviency and toadyism of some of his intimates; these private qualities, are apt to be carried into public life, and in consequence, he is not so much respected and admired by his brethren, and the public, as his great talents for his profession would lead one to expect. He is yet young, let us hope that he will 'change all that.'

Your obedient servant,
EDINENSIS."

### *Remarkable Case of Hemiplegia.*

" Francis Joseph, age 35 years, of sallow unhealthy complexion, a seaman on board an Indiaman, just arrived from England, was admitted into the General Hospital on the 27th of January, 1833, with hernia humoralis, which in a few days was relieved by free local bleeding and the exhibition of a solution of salts and tartar emetic to nausea and purging.

On the evening of the 6th of February, the patient called my attention to a sensation of numbness, and imperfect muscular power of the left upper extremity, and a drawing of the mouth to the right side, which he said had been gradually increasing during the two preceding days; the expression of his countenance was uncomfortable, and his pulse, though small, was quick and hard.

Bleeding to syncope, and a brisk mercurial purgative were prescribed.

At 6 A.M. the following day, I found him hemiplegic; the muscular power of the left half of the body being completely paralyzed. Sensation imperfectly continued, whilst the circulation and temperature of the affected parts was natural. He passed evacuations involuntarily, and the urine was retained. He was heavy and drowsy, but complained only of pain in the sciatic nerve; percussion in the course of the spinal column caused no uneasiness.

The head was shaved and cold applied, 30 leeches to the occiput, calomel purgatives and low diet prescribed.

8th February.—The paralytic symptoms unrelieved; frequent dark stools pass off in bed.

Thirty leeches to the cervical spine, and calomel purgatives continued. Catheter passed, and oz. iv. of fætid ammoniacal urine drawn.

9th.—No change for the better. Thirty leeches to the base of the head and cervical spine; calomel purgative and catheter passed.

10th.—He appears somewhat exhausted by depletion, and the smart action which has been kept up on the alimentary canal. Pulse small and quick, countenance anxious; paralytic state of the left half of the body continues complete. Mouth affected by mercury.

Blister from the back of the head to the sacrum. Pul. Jalap. comp. zi. at 7 A. M. Strychnine gr. $\frac{1}{4}$ every 3 hours —milk diet and sago. Urine drawn off.

11th & 12th.—The same remedies continued without advantage.

13th.—During last night he passed a small quantity of urine, and he can now retain the fæces until a bed-pan is placed under him; he can also raise the extremities from the bed. Remedies continued.

14th.—Passed 4 oz. of fætid urine in the night, and three voluntary stools; has more power over the extremities, and the features are less distorted.

16th.—Gradually improving under the same treatment; can now carry his arm up to his head.

21st.—Has been progressing since the last date. He can at this time walk, drawing the limb awkwardly after him, and raise the arm over the head; the muscles of the face have recovered their action, and the angle of the mouth is scarcely observed to incline to the right side when he speaks.

Medicine continued. Chicken diet.

March 13th.—Has perfect use of the whole of the lately paralyzed parts, but he feels weaker on that than on the opposite side.—Strychnine discontinued.

A mild aperient every moning.

April 23d.—Discharged perfectly cured, and in good health.

No tetanic or apparent nervous excitement was occasioned by the internal exhibition of Strychnine.

WALTER RALEIGH,
*General Hospital.*"

With these two extracts we must close our present notice of our new contemporary.

